# Distribution and possible function of galanin about headache and immune system in the rat dura mater

**DOI:** 10.1038/s41598-022-09325-3

**Published:** 2022-03-25

**Authors:** Kenichiro Shimazaki, Takehiro Yajima, Hiroyuki Ichikawa, Tadasu Sato

**Affiliations:** Division of Oral and Craniofacial Anatomy, Graduate School of Dentistry, Tohoku University, 4-1 Seiryo-machi, Sendai, 980-8575 Japan

**Keywords:** Neuroscience, Anatomy

## Abstract

Galanin (GAL) is a nociceptive transmitter or modulator in the trigeminal sensory system. In this study, GAL expression was investigated in the rat dura mater to demonstrate its possible function in headache using immunohistochemical techniques. The cerebral falx and cerebellar dura mater received abundant blood and nerve supply, and were significantly thicker compared to other portions in the cerebral dura mater. GAL-immunoreactivity was expressed by cell and nerve fiber profiles. Presumed macrophages and dendritic cells contained GAL-immunoreactivity, and co-expressed with CD11b-immunoreactivity. Many isolated and perivascular nerve fibers also showed GAL-immunoreactivity. In addition, GAL-immunoreactive nerve fibers were present in the vicinity of macrophages and dendritic cells with either GAL- or ED1-immunoreactivity. GAL-immunoreactive cells and nerve fibers were common in the cerebral falx and cerebellar dura mater and infrequent in other portions. And, GAL-immunoreactive nerve fibers usually co-expressed calcitonin gene-related peptide (CGRP)-immunoreactivity. In the trigeminal ganglion, a substantial proportion of sensory neurons innervating the dura mater contained GAL-immunoreactivity (mean ± SD, 3.4 ± 2.2%), and co-expressed CGRP-immunoreactivity (2.7 ± 2.1%). The present study may suggest that GAL is associated with nociceptive transduction or modulation in the dura mater. GAL also possibly plays a role in the immune mechanism of the dura mater.

## Introduction

The cranial dura mater is a hard membrane that surrounds the brain with the arachnoid mater and pia mater^1^. The dura mater and arachnoid mater keep the cerebrospinal fluid within the cranial cavity. The dural sinuses are supported by the cranial dura mater and send blood from the brain to the heart through the internal jugular vein^2^. The blood and nerve supply of the meninges is abundant in the dura mater but not in the arachnoid mater or pia mater^3^. In addition, the dura mater contains many macrophages and dendritic cells^4,5^. These cells may be able to protect the brain against foreign invaders and damages.

The dura mater is innervated from the trigeminal ganglion (TG). Previous retrograde tracing studies have demonstrated that sensory neurons innervating the dura mater have small to medium-sized cell bodies in the TG^6–8^. These neurons contain calcitonin gene-related peptide (CGRP), a putative nociceptive transmitter^9–11^. CGRP-containing TG neurons supply the dura mater with free nerve endings and perivascular endings^12^. In the trigeminal system, CGRP causes neurogenic inflammation^13^ such as vasodilation and plasma extravasation^14^. A previous in vitro study using the RAW264.7 cell line has demonstrated that CGRP can also regulate the synthesis of cytokines in macrophages^15^. Thus, CGRP-containing TG neurons may contribute to nociceptive transduction and immune response in the dura mater.

Galanin (GAL), another member of the neuropeptide family, is distributed in the central and peripheral nervous systems^16–19^. This peptide can modulate or inhibit action potentials in brain and spinal cord neurons. In the sensory ganglion, GAL is mainly expressed by small neurons^20–22^. Previous double immunofluorescence studies have also demonstrated the co-expression of GAL and CGRP in the TG^23,24^. Similar to CGRP-containing TG neurons, GAL-containing TG neurons transduce nociceptive information from cranial structures to the brainstem^22,25^. Our previous study also demonstrated that GAL and its receptors in the TG respond to nerve transection and suggested that they protect TG neurons from damage through their autocrine and paracrine mechanisms^22^. In addition, GAL is expressed by macrophages derived from peripheral blood mononuclear cells and is shown to affect their activity by modulating some chemokines and cytokines^26,27^. Thus, we hypothesize that GAL in the dura mater functions in the nociceptive transmission and defense mechanism by the immune system.

Determining the distribution and origin of GAL and its relationship with immune cells in the dura mater may facilitate our understanding of GAL function in the nociceptive and immune defense mechanisms. However, little is known about the distribution or function of GAL in the dura mater. In the present study, the distribution of GAL was examined in the rat dura mater. The relationship between GAL- and CGRP-containing profiles was also investigated by a double immunofluorescence method. In addition, a retrograde tracing method was performed to determine the origin of GAL-containing nerve fibers in the dura mater.

## Results

### Structure of the dura mater

**Figure 1:**
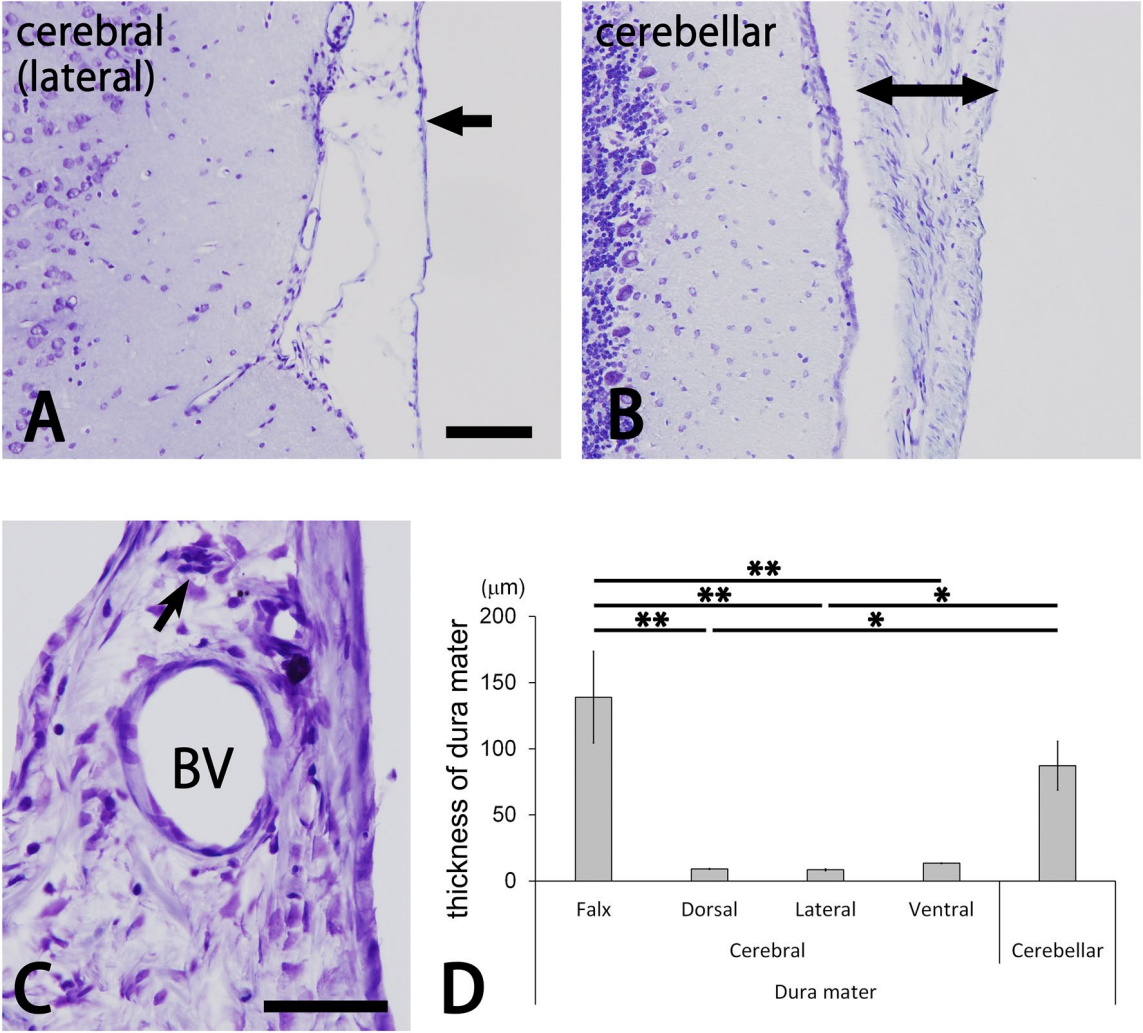
Photomicrographs for cresyl violet-stained cerebral and cerebellar dura mater (**A**–**C**). The lateral portion of the cerebral dura mater is thin (arrow in **A**), whereas the cerebellar dura mater is very thick (right and left arrows in **B**). The thick dura mater (**C**) contains a large blood vessel (BV) and nerve bundle (arrow). Bars = 100 µm (A) and 50 µm (C). Panels (**A**) and (**B**) are at the same magnification. Bar graphs indicate the thickness of the cerebral and cerebellar dura mater (**D**). The cerebral falx and cerebellar dura mater were significantly thicker than other portions of the cerebral dura mater (Tukey’s test *p < 0.05; **p < 0.01). Data were obtained from three sections of the cerebral falx; three to six sections of the dorsal, lateral and ventral portions of the cerebral dura mater; and two sections of the cerebellar dura mater in each of the four animals.

As described previously, the dura mater had outer and inner layers^28^. The outer layer consisted of thin fibrous tissues containing simple squamous cells. Meanwhile, the inner layer of the dura mater was of various thicknesses (Fig. 1). In the cerebral falx and cerebellar dura mater, as well as in the dura mater around the olfactory bulb, the deep layer was thick and contained large blood vessels and nerve bundles (Fig. 1B,C). In other portions of the dura mater, the deep layer was thin and mostly free of them (Fig. 1A). As a result, the thickness of the cerebral falx and cerebellar dura mater was significantly larger than that of other portions in the cerebral dura mater (Fig. 1D).

### GAL-positive cell and nerve fiber profile

**Figure 2:**
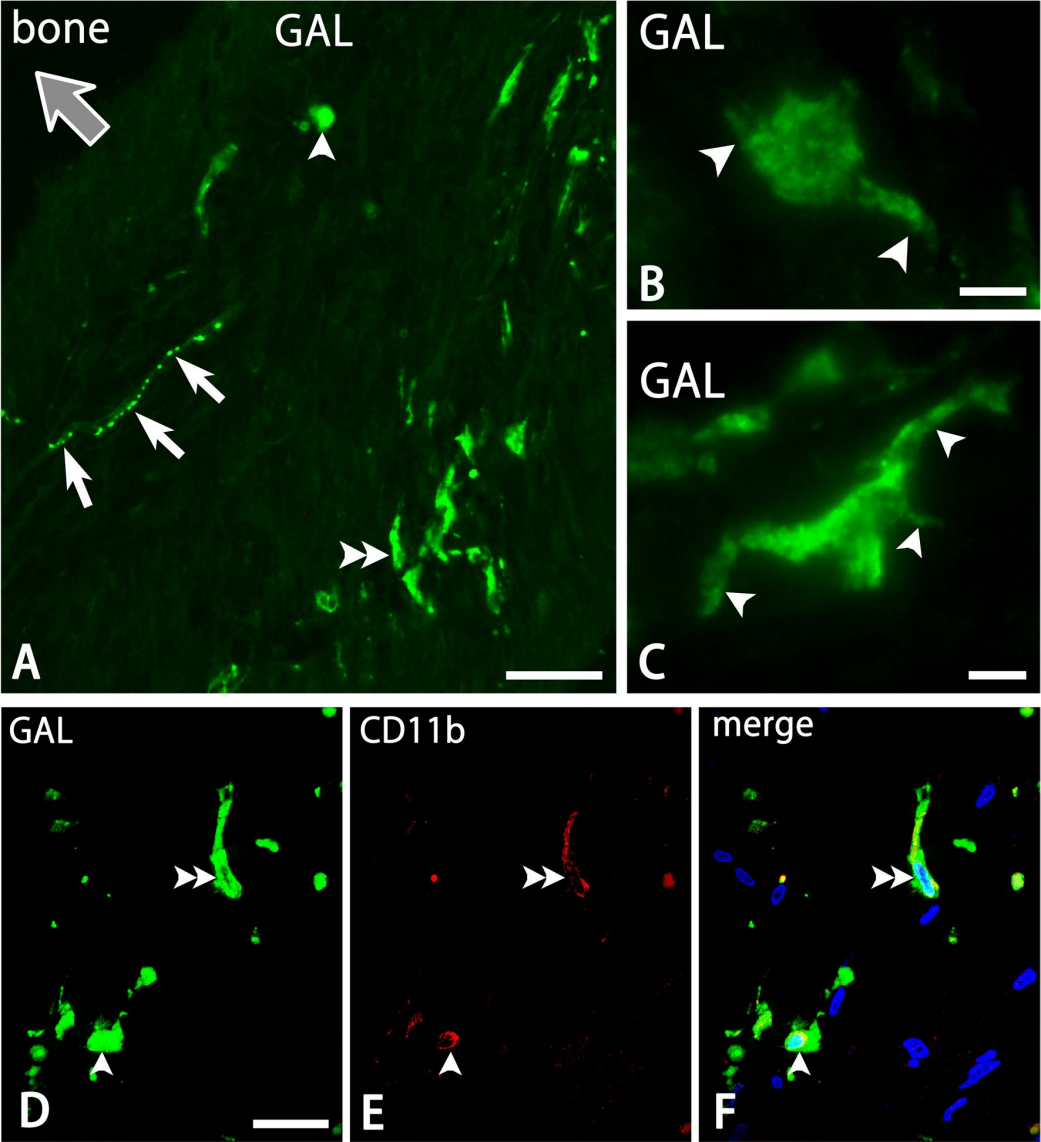
Photomicrographs for GAL (**A**–**D**,**F**), CD11b (**E**,**F**) and DAPI (**F**) in the cerebellar tentorium by a conventional fluorescence microscope (**A**–**C**) and a confocal laser scanning microscope. Panels (**D**–**F**) are from the same field of views. GAL-immunoreactivity is expressed by round (an arrowhead) and oblong cells (a double arrowhead), and varicose nerve fibers (arrows) in the thick dura mater (**A**). Gray arrows indicate the direction toward the bone (**A**). Panels (**C**) and (**D**) show round and oblong types, respectively, of GAL-ir cells with some processes (arrowheads). GAL- and CD11b-immunoreactivity are co-expressed by round (arrowheads) and oblong (double arrowheads) cells (**D**–**F**). Bars = 50 µm (**A**), 20 µm (**D**) and 10 µm (**B**,**C**). Panels (**D**–**F**) are at the same magnification.

**Figure 3:**
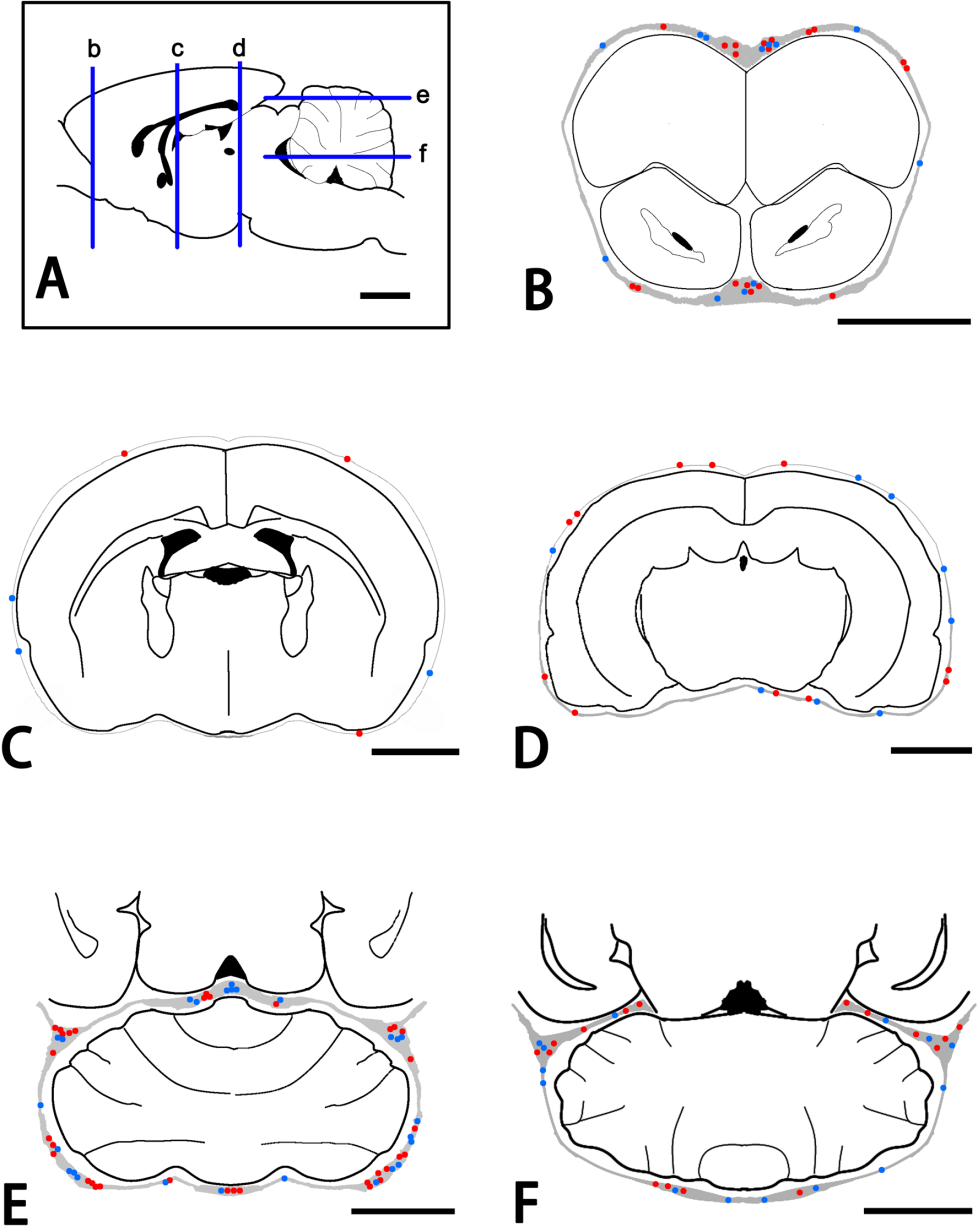
Schematic drawings for the distribution of GAL-ir cells in the dura mater. Panels (**B**–**F**) indicate the frontal (**B**–**D**) and horizontal (**E**,**F**) sections of the brain obtained from the cross sections of b, c, and d, and e and f, respectively. Red and blue circles indicate round and oblong cells with GAL-immunoreactivity, respectively. Bars = 3 mm (**A**–**F**).

GAL-immunoreactivity was expressed by cell and nerve fiber profiles in the dura mater (Fig. 2A). Morphologically, GAL-immunoreactive (-ir) cells were divided into two types: round and oblong types. The round type had round and oval cell bodies, as well as fine and short processes with slight ramifications (Fig. 2B). In contrast, the oblong type showed long-shaped cell bodies with many thick and ramified processes (Fig. 2C). The oblong type cells (average size ± SD = 124.3 ± 51.0 µm^2^; range = 36.3–281.9 µm^2^; n = 112) were larger than round type cells (101.8 ± 48.7 µm^2^; 28.9–246.8 µm^2^; n = 147). Both types of GAL-ir cells were usually distributed in the deep layer of the dura mater. They were also abundant in the cerebral falx and cerebellar dura mater, and around the olfactory bulb (Fig. 3). A double immunofluorescence method demonstrated that both round and oblong types of GAL-ir cells were also immunoreactive for CD11b (Fig. 2D–F).

**Figure 4:**
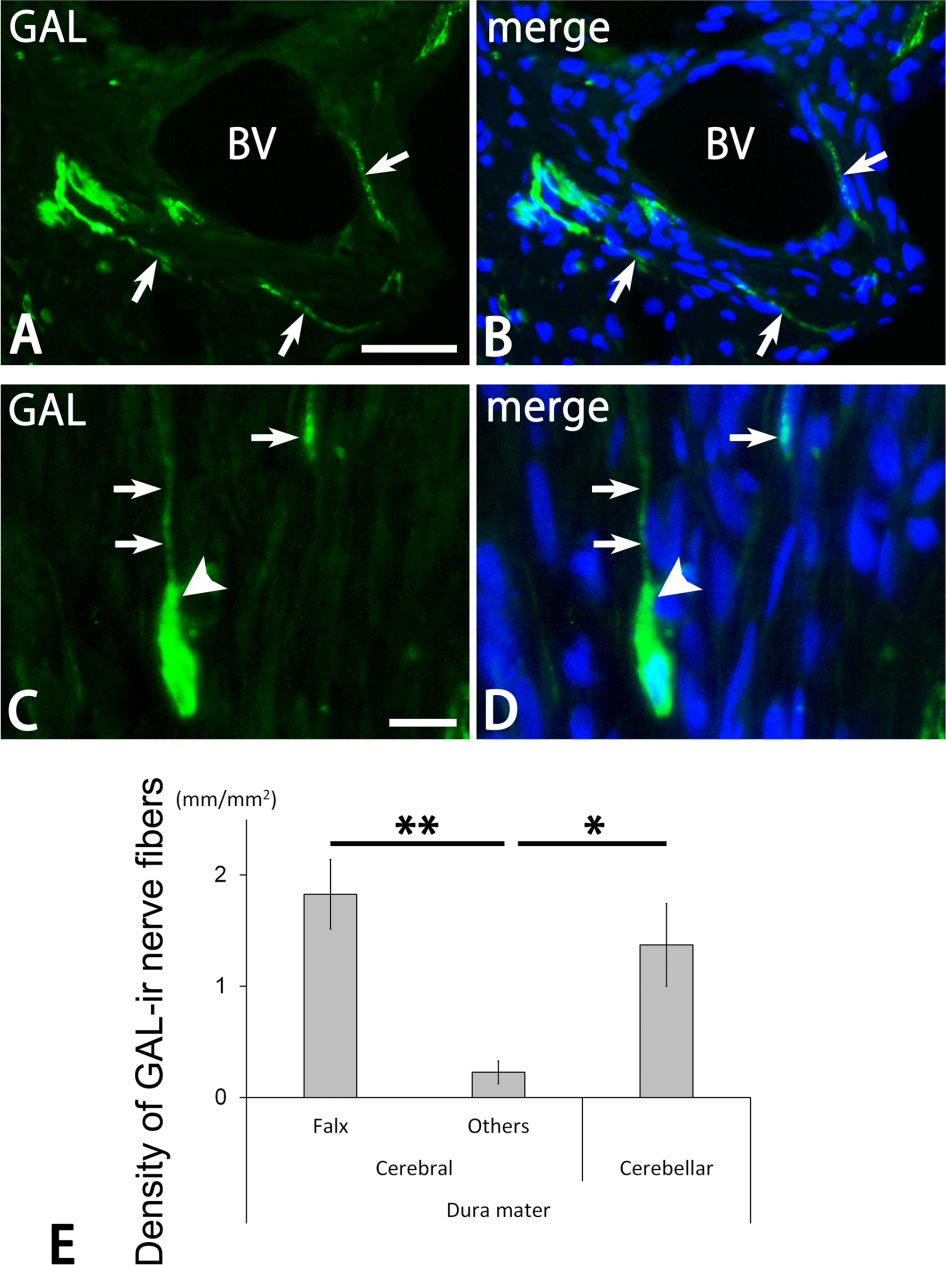
Photomicrographs for GAL (**A**–**D**) and DAPI (**B**,**D**) in the dura mater by a conventional fluorescence microscope. Bar graphs indicating the density of GAL-ir nerve fibers in the dura mater. Perivascular nerve fibers with varicosities (arrows in **A**,**B**) contain GAL-immunoreactivity. GAL-ir nerve fibers (arrows in **C**,**D**) are also detected in the vicinity of oblong (arrowheads in **C**,**D**) GAL-ir cells. *BV* blood vessel. Bar = 50 µm (**A**) and 20 µm (**C**). Panels (**A**,**B**) and (**C**,**D**) are at the same magnifications, respectively. The density of GAL-ir nerve fibers in the cerebral falx and cerebellar dura mater is significantly higher than in other portions of the cerebral dura mater (Tukey’s test, **p < 0.01; *p < 0.05). The data were obtained from three sections of the cerebral falx, 12 sections of other portions of the cerebral dura mater, and two sections of the cerebellar dura mater in each of the four animals.

**Figure 5:**
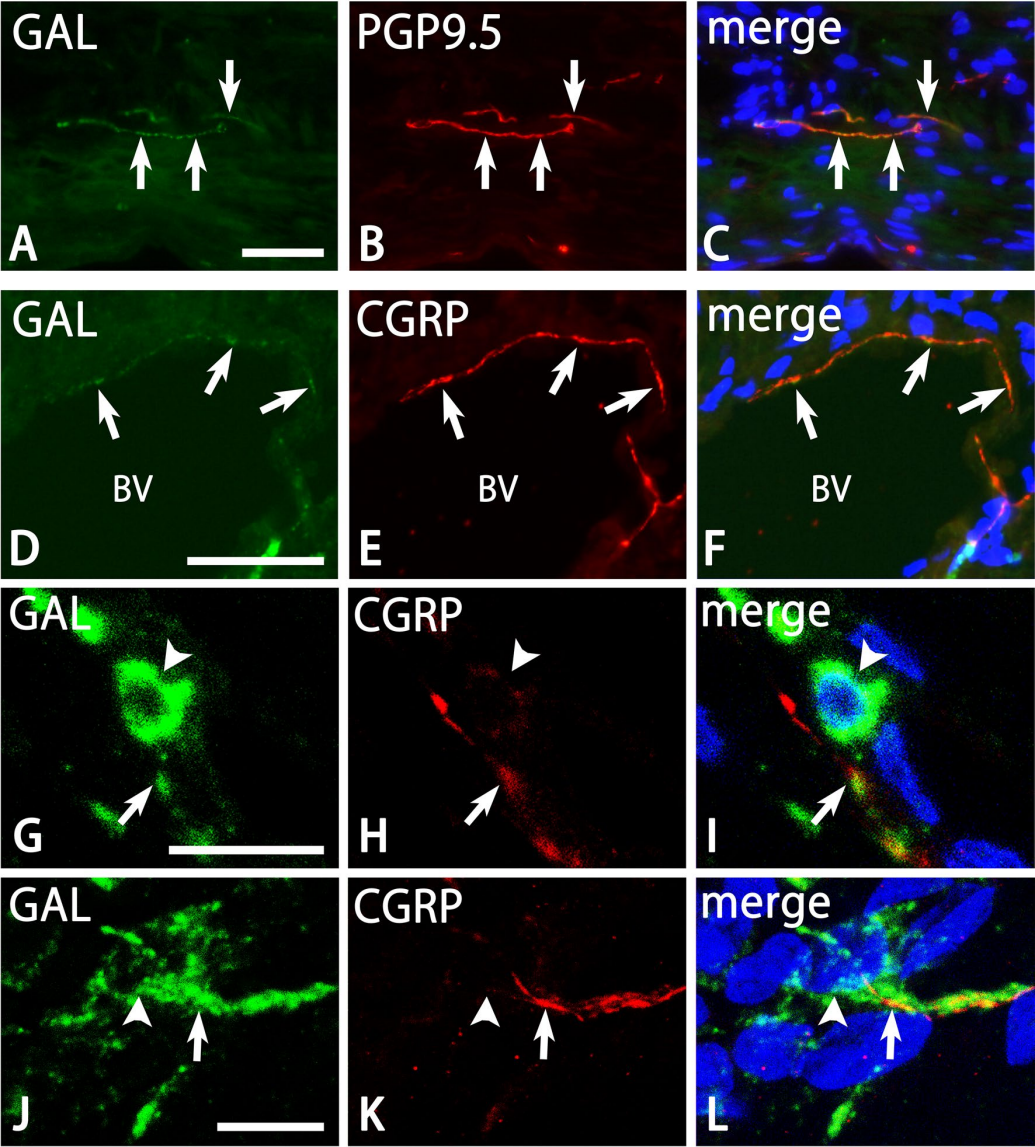
Photomicrographs for GAL (**A**,**D**,**G**,**J**), PGP9.5 (**B**), CGRP (**E**,**H**,**K**), and merged and DAPI (**C**,**F**,**I**,**L**) in the dura mater by a conventional fluorescence (**A**–**F**) and a confocal laser scanning (**G**–**L**) microscope. Panels (**A**–**C**), (**D**–**F**), (**G**–**I**), and (**J**–**L**) are from the same fields of view, respectively. Panels (**J**–**L**) were obtained from 13 sections, respectively, at 0.5 µm interval. Isolated (**A**–**C**) and perivascular (**D**–**F**) nerve fibers, and nerve fibers apposed to cells (**G**–**L**) with GAL- (**A**,**D**,**G**,**J**), PGP9.5- (**B**,**C**), and CGRP-immunoreactivity (**E**,**F**,**H**,**I**,**K**,**L**) show similar distributions. Arrowheads in (**G**–**I**) and (**J**–**L**) indicate round and oblong types of GAL-ir cells, respectively. Bars = 50 µm (**A**,**D**) and 10 µm (**G**,**J**). Panels (**A**–**C**), (**D**–**F**), (**G**–**I**), and (**J**–**L**) are at the same magnifications, respectively.

**Figure 6:**
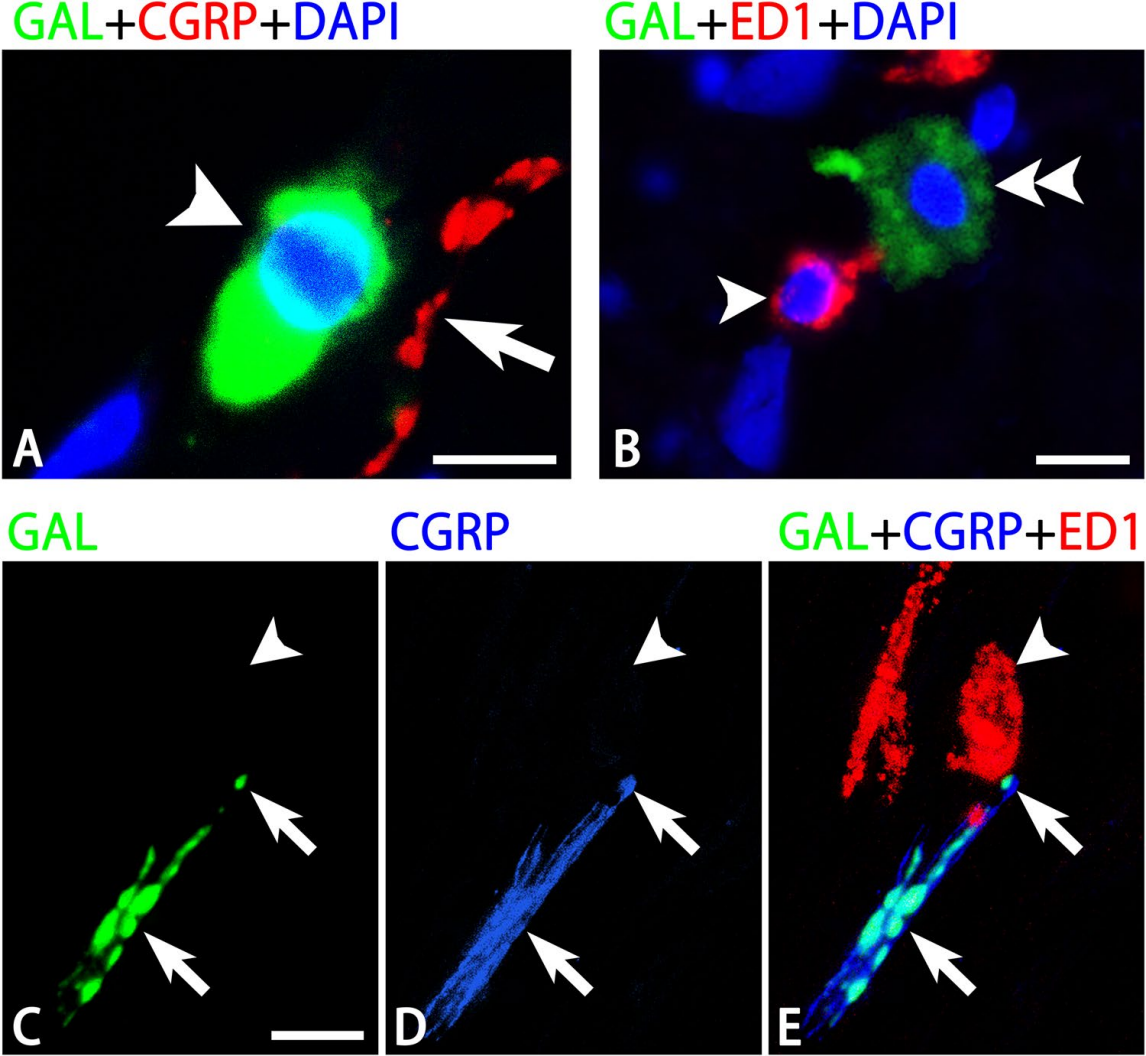
Photomicrographs for GAL (**A**,**B**,**C**,**E**), CGRP (**A**,**D**,**E**), ED1 (**B**,**E**) and DAPI (**A**,**B**) in the dura mater by a confocal laser scanning microscope. Panels (**C**–**E**) are from the same field of view. CGRP-ir varicose fibers (an arrows in **A**) in the vicinity of a GAL-ir cell (an arrowhead in **A**) often lack GAL-immunoreactivity. A GAL-ir cell (a double arrowhead in **B**) is also detected in the vicinity of ED1-ir cells without GAL-immunoreactivity (an arrowheads in **B**). A nerve fiber that co-expresses GAL- and CGRP-immunoreactivity (arrows in **C**–**E**) are observed in the vicinity of an ED1-ir cell (arrowheads in **C**–**E**). Bars = 10 µm (**A**–**C**). Panels (**C**–**E**) are at the same magnification.

Many GAL-ir nerve fibers were detected in the dura mater (Figs. 2, 4, 5). These nerve fibers showed fine varicose or smooth appearance and were usually located within thin and thick nerve bundles (Figs. 4, 5). Some of them separated from the nerve bundles and slightly ramified as isolated nerve endings with varicosities. GAL-ir nerve fibers were also detected in the vicinity of large blood vessels in the deep layer of the dura mater (Fig. 4A,B). In addition, GAL-ir nerve fibers were apposed to GAL-ir cells (Fig. 4C,D). Some GAL-ir varicose fibers ran toward round and oblong GAL-ir cells and occasionally made a close apposition to them. The density of GAL-ir nerve fibers in the cerebral falx and cerebellar dura mater was significantly higher than in other portions of the cerebral dura mater (Fig. 4E). In a double immunofluorescence analysis, the distribution pattern of GAL-ir nerve fibers was similar to that of PGP9.5- and CGRP-ir nerve fibers in the dura mater (Fig. 5). However, PGP9.5- and CGRP-ir nerve fibers without GAL-immunoreactivity were also abundant within large nerve bundles and apposed to blood vessels. Some CGRP-ir nerve fibers in the vicinity of GAL-ir cells were free of GAL-immunoreactivity (Fig. 6A).

ED1-immunoreactivity was expressed by several round and oblong cells with many dotted processes in the dura mater. Similar to GAL-ir cells, ED1-ir cells were abundant in the deep layer of the cerebral falx and cerebellar dura mater, as well as of the dura mater around the olfactory bulb. ED1-ir cells in the dura mater were mostly free of GAL-immunoreactivity. However, some GAL-ir cells were detected in the vicinity of ED1-positive cell bodies and processes (Fig. 6B). In addition, many GAL-ir nerve fibers, which were isolated nerve endings in single GAL stain, were seen close to round ED1-ir cells. Both round and oblong types of ED-ir cells appeared to be innervated by GAL-ir nerve fibers, mostly co-expressing CGRP-immunoreactivity (Fig. 6C–E).

### Retrograde tracing analysis

**Figure 7:**
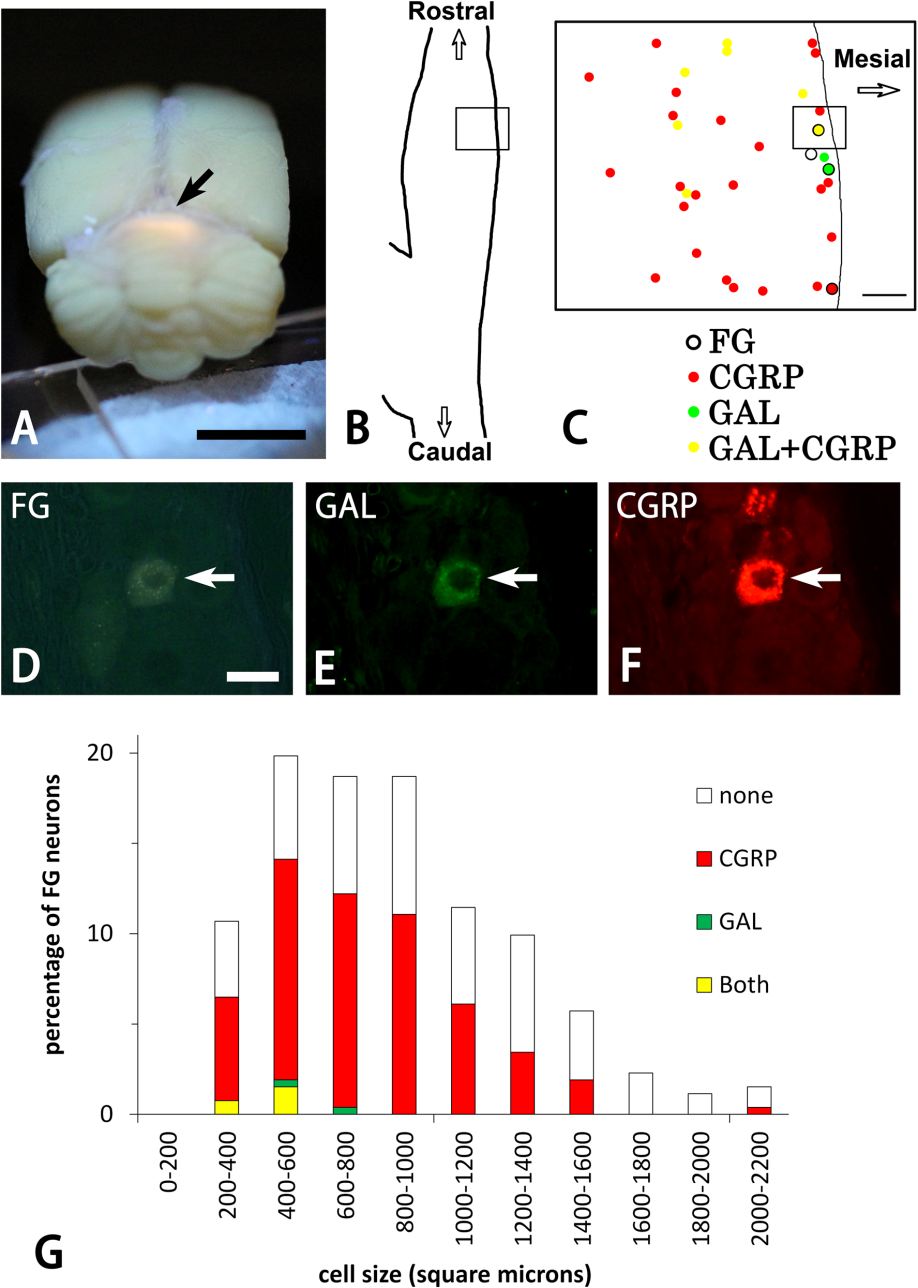
Photo for FG in the brain (**A**), schematic drawings of TG (**B**,**C**), and photomicrographs for FG (**D**), GAL (**E**), and CGRP (**F**) in the TG by a conventional fluorescence microscope (**A**,**D**–**F**). FG fluorescence is detected in the dorsal portion of the cerebellar tentorium (arrow in **A**). Squares in (**B**) and (**C**) show the areas of panels (**C**) and (**D**–**F**), respectively. FG-positive neurons are mainly located in the ophthalmic region of the TG (**B**,**C**). These neurons contain GAL- and/or CGRP-immunoreactivity (**C**). Panels (**D**–**F**) are from the same field of view. A TG neuron retrogradely labeled with FG from the cerebellar dura mater (arrow in **D**) contains both GAL- (arrow in **E**) and CGRP-immunoreactivity (arrow in **F**). Bars = 5 mm (**A**) and 20 µm (**D**). Panels (**D**–**F**) are at the same magnification. Bar graphs showing the cell size spectrum of FG-positive TG neurons with and without GAL- and CGRP-immunoreactivity. Data are obtained from 262 FG-positive TG neurons in four animals.

The retrograde tracing method demonstrated that fluorogold (FG)-positive neurons were present in the rostral 2/3 of the TG. A substantial proportion (mean ± SD, 3.4 ± 2.2%, n = 4) of FG-positive TG neurons were GAL-ir. Moreover, half (58.7 ± 10.0%, n = 4) of FG-positive TG neurons showed CGRP-immunoreactivity (Fig. 7). As reflected by few FG-positive TG neurons with GAL-immunoreactivity, 95.6 ± 1.3% (n = 4) of FG-positive TG neurons with CGRP-immunoreactivity lacked GAL-immunoreactivity. However, FG-positive neurons with GAL-immunoreactivity mostly co-expressed CGRP-immunoreactivity (83.3 ± 19.2%, n = 4). Data were obtained from 262 FG-positive neurons in four animals.

In terms of size, FG-positive neurons were mostly small to medium-sized (857.6 ± 50.6 µm^2^, n = 4). Thirty-one % (80/262) and 48.9% (128/262) of total FG-positive neurons had small (< 600 µm^2^) and medium-sized cell bodies (600–1200 µm^2^), respectively. Large FG-positive neurons were relatively infrequent in the TG (> 1200 µm^2^, 20.6%, 54/262). FG-positive TG neurons with small to medium-sized cell bodies contained GAL- (499.8 ± 53.8 µm^2^, n = 4) and CGRP-immunoreactivity (762.8 ± 28.3 µm^2^, n = 4). All FG-positive neurons which co-expressed GAL- and CGRP-immunoreactivity had small cell bodies (Fig. 7G). Some FG-positive neurons were also scattered in the superior cervical ganglion. However, the sympathetic neurons were immuno-negative for GAL and CGRP (photo not shown).

## Discussion

The present immunohistochemical study demonstrated that GAL was expressed by the cell and nerve fiber profiles in the dura mater. GAL-positive cells had round and oblong cell bodies. The present finding that GAL-positive cells co-expressed CD11b-immunoreactivity suggests the possibility that those cells include polymorphonuclear and mononuclear cells. However, GAL-positive cells had multiple processes in the dura mater. Because polymorphonuclear cells have round cell bodies and lack processes, they are probably immune cells differentiated from mononuclear cells^29^. The morphology of their processes also appeared to be different between the two types of GAL-ir cells. The round type had fine and short processes whereas the oblong type had thick, long, and ramified processes. Co-expressing GAL and CD11b in these cell bodies, their morphology suggests that round and oblong GAL-ir cells were identical to macrophages and dendritic cells, respectively. This can be supported by the previous finding that macrophages originating from human blood mononuclear cells showed GAL mRNA expression^27^. Because GAL-containing cells were abundant in the cerebral falx and cerebellar dura mater, GAL is possibly associated with the immune system in those regions.

In the dura mater, GAL-containing nerve fibers were seen in the vicinity of GAL-containing cells, which are presumed to be macrophages and dendritic cells. Many isolated nerve endings were also GAL-ir. However, in a double immunofluorescence analysis, GAL-containing nerve fibers were present around ED1-positive cells. Most ED1-positive cells were free of GAL-immunoreactivity and had round and oblong cell bodies with processes. ED1 is also known to be a marker for macrophages and dendritic cells^30,31^. These findings indicate that GAL-containing nerve fibers are probably located around macrophages and dendritic cells with and without GAL-immunoreactivity. In addition, GAL- and ED1-positive cells were occasionally close to each other. A previous study has demonstrated that macrophages have GAL receptors and that exogenous GAL regulates the expression of some chemokines, transforming growth factor β, interleukin-10, and interleukin-1Ra in macrophages for their differentiation, polarization, and anti-inflammatory function^27^. The occurrence or distribution of GAL receptors have not been reported in the dura mater. Nonetheless, the present findings about distribution of GAL-positive nerve fibers suggest that GAL receptors are present in blood vessels and GAL-positive and -negative cells. A previous data that GAL3 receptor was abundant in cutaneous vasculatures and immune cells, including macrophages, supports this finding^32,33^. Therefore, GAL secreted from cell and nerve fiber profiles within the dura mater may affect the surrounding immune cells through autocrine, paracrine, and neural mechanisms in the dura mater.

The present double immunofluorescence study also showed co-expressed GAL and CGRP in nerve fibers around blood vessels and the presumed macrophages and dendritic cells. The present retrograde tracing analysis demonstrated that these nerve fibers originate from the TG. Previous studies have suggested that CGRP in TG neurons is involved in vascular headaches^34–36^. CGRP is also considered to contribute to vasodilation via nociceptive transduction from the head and other regions. In addition, a previous study has demonstrated that increased GAL mRNA and decreased CGRP mRNA in sensory neurons are induced by the capsaicin treatment, which can cause vasodilation and extravasation through CGRP and substance P release from sensory terminals^37,38^. Thus, GAL and CGRP functions may be different in the nociceptive transmission and vascular regulation. However, the present co-expression data on GAL and CGRP in nerve fibers within the dura mater suggest that neural GAL is also associated with migraine. On the other hand, CGRP is known to inhibit the synthesis of tumor necrosis factor-alpha in lipopolysaccharide-stimulated macrophages. Exogenous CGRP also causes increased interleukin-6 release and decreased interleukin-12 in macrophages^39^. In the dura mater, the function of these neuropeptides remains unclear. Nonetheless, co-expressed GAL and CGRP in nerve fibers around the presumed macrophages and dendritic cells suggest that the neuropeptides released from the same nerve terminal affect the synthesis of cytokines and chemokines. GAL and CGRP also appear to have opposite functions on macrophages. The expression of several cytokines and chemokines is upregulated by CGRP and downregulated by GAL^15,26,27^ GAL may also be able to inhibit CGRP function in macrophages and dendritic cells within the dura mater.

Coexistence of GAL and CGRP has been previously demonstrated in small sensory neurons of the human dorsal root ganglion (DRG)^37^. In addition, the proportion of sensory neurons expressing GAL mRNA decreases in the DRG of a patient with migraine-like pain, compared to the un-lesioned dorsal root ganglion. In contrast, GAL mRNA-positive neurons increase in the DRG of a patient with herpes zoster^40^. In the human TG, GAL function about headache has not been previously demonstrated. However, similar to the human DRG, it is possible that GAL expression changes in the human TG during headache and that GAL contributes to nociceptive transmission from the human head. Although the functional relationship between GAL and CGRP in the headache and immune system remains unclear, there appears to be neuro-immunological communication via sensory GAL and CGRP. In this study, CGRP-containing nerve fibers without GAL-immunoreactivity were also detected in the vicinity of GAL-positive cells. Noxious stimulation can activate TG neurons and release CGRP with and without GAL from their peripheral nerve endings. In the dura mater, these peptides may affect blood flow, differentiation, activity and cytokine secretion of immune cells, and thereby immune responses including inflammation. In the brainstem. released GAL and CGRP probably modulate activity of secondary nociceptive neurons and control nociceptive transduction from the head. Further studies using physiological and pharmacological methods will be necessary to know the functional role of GAL on the nociceptive transmission and immune system in the dura mater.

## Materials and methods

### Experimental animals

Twelve male Wistar rats (7 weeks old, 200–250 g) were used in this study. They were purchased from CLEA Japan, Inc. (Japan). Eight and 4 rats were used for tissue preparation and retrograde tracing study, respectively.

All animal experiments were performed according to The Guidelines for Care and Use of Laboratory Animals in Tohoku University, and the Japanese Government Notification on Feeding and Safekeeping of Animals (No. 6), which are in line with ARRIVE guidelines^41^. All experiments were performed after being reviewed and approved by Institutional Laboratory Animal Care and Use Committee of Tohoku University (2020DnA-040).

### Tissue preparation

Eight male Wistar rats were used to determine the distribution of GAL-ir nerve fibers in the dura mater. They were anesthetized with isoflurane (Fujifilm Wako Pure Chemical Corporation, Japan) until respiration was markedly suppressed and transvascularly perfused with 50 mL saline followed by 500 mL Zamboni fixative^42^. The brain with the meninges was dissected and immersed in the same fixative. Then, the samples were immersed overnight in a phosphate-buffered saline containing 20% sucrose and frozen with dry ice. The whole brain was frontally and serially sectioned at 8 µm and thaw-mounted on silane-coated glass slides (n = 4). The caudal 1/3 portion of the brain, including the brainstem and cerebellum, was also horizontally sectioned in four animals. The 480-µm interval sections of the brain were stained with 0.2% cresyl violet solution (Sigma-Aldrich, USA) and subjected to immunohistochemistry to demonstrate the structure of the dura mater and the distribution of GAL and other substances within it.

### Immunohistochemistry

To analyze the expression of GAL and its relationship to CD11b and ED1 (markers for macrophages and dendritic cells), protein gene product 9.5 (PGP9.5, a marker for neural elements), and CGRP, single, double and triple immunofluorescence methods were used for the rat dura mater (Table 1). The sections were incubated with appropriate secondary antibodies (Table 1) for the immunofluorescence and tyramide signal amplification (TSA) methods (Perkin Elmer, USA).

**Table 1:** Primary and secondary antibodies used in this study.

Antigen	Species	Source	Catalog No	Dilution
CD11b B cell, macrophage, and dendritic cell marker^43^	Rabbit	Abcam, UK	ab133357	1:300
CD11b B cell, macrophage, and dendritic cell marker^43^	Mouse	Serotec, UK	MCA275G	1:100
CGRP Nociceptive transmitter^44^	Guinea pig	Peninsula Laboratories, USA	T-5053	1:1000–2000
ED1 Macrophage and dendritic cell marker^30,31^	Mouse	Serotec, UK	MCA341GA	1:100
Galanin	Rabbit	Peninsula Laboratories, USA	T-4334	1:1000 for immunofluorescence 1:10,000 for the TSA method
PGP9.5 Neural elements marker ^45^	Rabbit	UltraClone Ltd., UK	RA 95101	1:2000

Secondary antibody	Species	Source	Dilution	
Aminomethylcoumarin-conjugated anti-guinea pig IgG for CGRP	Donkey	Jackson ImmunoResearch Labs., USA	1:100	
Biotinylated anti-rabbit IgG for galanin	Goat	Vector laboratories, USA	1:200	
Fluorescein isothiocyanate-conjugated anti-rabbit IgG for galanin	Donkey	Jackson ImmunoResearch Labs., USA	1:100	
Rhodamine red™-X-conjugated anti-guinea pig IgG for CGRP	Donkey	Jackson ImmunoResearch Labs., USA	1:300	
Rhodamine red™-X-conjugated anti-mouse IgG for ED1	Donkey	Jackson ImmunoResearch Labs., USA	1:300	
Rhodamine red™-X-conjugated anti-rabbit IgG for CD11b and PGP9.5	Donkey	Jackson ImmunoResearch Labs., USA	1:300	

For GAL immunofluorescence, the sections were incubated with 80% methanol and 0.3% hydrogen peroxide solution for 30 min to prevent endogenous peroxidase reaction. Sections were incubated with tris-NaCl-blocking buffer for 60 min and rabbit anti-GAL serum overnight. Samples were immersed in biotinylated goat anti-rabbit IgG serum (Vector, 1:200) for 60 min and avidin–biotin-complex prepared by VECTASTAIN Elite ABC Reagent (Vector) for 60 min. Then, sections were incubated with fluorescence-tyramide working solution for 10 min. After checking for GAL immunofluorescence, double and triple immunofluorescence were performed to demonstrate the relationship of GAL to CD11b, ED1, and CGRP. The GAL-stained sections were incubated with mouse anti-CD11B or anti-ED1 IgG and guinea pig anti-CGRP serum. The sections were further incubated with rhodamine red™-X or aminomethylcoumarin-conjugated donkey antiserum against guinea pig IgG or mouse IgG serum. For GAL and CD11b or PGP9.5, GAL-stained sections were incubated with rhodamine red™-X-conjugated donkey anti-rabbit IgG serum. After confirming no immunofluorescence by the secondary antiserum and rabbit anti-GAL serum, the sections were incubated with rabbit anti-CD11b and anti-PGP9.5 serum, followed by rhodamine red™-X-conjugated donkey anti-rabbit IgG serum. Then, 4′,6-diamidino-2-phenylindole (DAPI, Sigma-Aldrich) was applied to some immunofluorescence-stained sections as a nuclear counterstain.

All procedures were conducted at room temperature. Sections were viewed with a conventional fluorescence microscope (ECLIPSE 80i, Nikon, Japan) or a confocal laser scanning microscope (A1+, Nikon). The specificity of antibodies against GAL, CGRP, CD11b, ED1, and PGP9.5 has been described elsewhere^22,46–50^. To check inappropriate reaction with the primary and secondary antibodies, TSA and double immunofluorescence methods were performed without either one or all of primary antibodies. The control sections showed only appropriate fluorescence. In addition, CD11b antibody was absorbed by the respective molecule (20 µg/ml). CD11b fluorescence cannot be detected in the absorption control.

### Morphometric analysis

For the morphometric data, the brain was divided into three portions: the rostral 1/3, middle 1/3, and caudal 1/3 portions. Three to twelve sections were randomly selected from each of these portions in each of the four animals. The total length of the GAL-ir nerve fibers within the dura mater was measured in each of the various regions with a personal computer (Hewlett-Packard, Japan), a digital pencil (Wakom, Japan), and a digital tracing tablet (Wakom) by freehand drawing (Lumina Vision program, Mitani Corporation, Japan). Data about the cerebral falx, other cerebral portions, and cerebellar dura mater were obtained from 1, 3, and 14 digital images (each image size = 660 µm × 820 µm), respectively, in each section. The proportion of their length to the area of each region was calculated for immunoreactive density^51–53^. The average of such immunoreactive density was recorded for each animal. Difference among several regions was examined by analysis of variance and Tukey’s test using the JMP^®^ 16 statistical software (SAS Institute Inc., Cary, NC, USA).

### Retrograde tracing analysis

To demonstrate the origin of GAL-ir nerve fibers in the dura mater, four male rats were used. Under deep anesthesia by i.p. injection with a mixture of 0.15 mg/kg medetomidine (Nippon Zenyaku Kogyo Co., Ltd., Japan), 2.0 mg/kg midazolam (Astellas Pharma Inc., Japan), and 2.5 mg/kg butorphanol (Meiji Seika Pharma Co., Ltd., Japan), the animal was put on the operative table, and the midline incision of the cranial skin was made at the back of its head. A small hole (diameter = 3 mm) was made on the lambda of the cranial bone with a dental round bar (diameter = 0.8 mm, Shofu inc., Japan). After washing out small bone fragments with saline solution, the excess saline solution, blood, and exudate fluid were wiped out with a dry cotton. Then, a crystal of FG (Fluorochrome, USA) with a diameter of 0.5 mm was placed on the middle portion of the tentorium cerebelli. The fluorescent tracer on the dura mater was sealed with α-cyanoacrylate (Toagosei Co., Ltd., Japan). The hole on the cranial bone was covered with the skin, and the skin was subsequently stitched with a 4–0 silk suture (Natsume Seisakusho Co., Ltd, Japan)^7^.

After 3 days, the animals were re-anesthetized with the same mixture, and transvascularly perfused with Zamboni fixative^42^. The TG and superior cervical ganglion were dissected and frozen-sectioned at 8 µm thickness. The sections of the TG were processed for co-expression of GAL and CGRP as described above. To determine the proportion of GAL- and CGRP-ir neurons among dura mater neurons, all FG-positive neurons were analyzed in every fifth section of the serial sections of the TG. For cell size analysis of GAL- and CGRP-ir neurons innervating the dura mater, the cross-sectional area of FG-positive cell bodies containing nuclear profiles was measured (Lumina Vision)^54^.
